# Rituximab for retroperitoneal fibrosis due to IgG4-related disease: A case report and literature review 

**DOI:** 10.5414/CNCS109321

**Published:** 2018-04-27

**Authors:** Mohammad Almeqdadi, Mohammed Al-Dulaimi, Aleksandr Perepletchikov, Kevin Tomera, Bertrand L. Jaber

**Affiliations:** 1 Department of Medicine,; 2 Division of Nephrology, Department of Medicine,; 3 Division of Urology, Department of Surgery, St. Elizabeth’s Medical Center,; 4 Department of Medicine, Tufts University School of Medicine,; 5 Department of Pathology, St. Elizabeth’s Medical Center, and; 6 Department of Neurology, Tufts Medical Center, Boston, MA, USA

**Keywords:** IgG4-related disease, retroperitoneal fibrosis, acute kidney injury, hydronephrosis, rituximab

## Abstract

Retroperitoneal fibrosis (RPF) is a progressive fibroinflammatory disease that can be complicated by urinary obstruction. RPF can be the only manifestation of IgG4-related disease (IgG4-RD). Treatment of IgG4-related RPF is challenging and mostly consists of long-term glucocorticoids leading to significant side effects and treatment intolerance. Recent exploration of the role of rituximab as a B-cell depleting therapy in the treatment of IgG4-RD provides therapeutic potential as a well-tolerated alternative to glucocorticoids. We present a case of IgG4-related RPF for which rituximab was instituted as a steroid-sparing treatment strategy. Following 4 doses, kidney function partially recovered, and the disease went into remission. We discuss the potential merit of rituximab for the treatment of patients with IgG4-related RPF.

## Introduction 

Retroperitoneal fibrosis (RPF) is a rare fibroinflammatory disorder that affects the soft tissue in the retroperitoneal space, originally described in 1948 by Dr. John Ormond [1] as a cause of extraluminal bilateral ureteral obstruction. RPF was later recognized to affect perivascular areas surrounding the aorta and common iliac vessels, and could extend to include the pancreas, ureters, and the renal arteries [2]. Although there are no standardized diagnostic criteria for RPF, it has been divided into idiopathic forms, where no cause has been identified, and secondary forms due to, for example, malignant diseases [2]. Approximately 30% of RPF cases are secondary to medications or malignancies [2, 3]. One of the rare secondary forms of RPF is due to IgG4-related disease (IgG4-RD), which, in a recent study, was suspected to be the cause of 60% of what was previously believed to be idiopathic RPF [4]. 

IgG4-related disease is an immune-mediated systemic disorder that was first described as a systemic disease in 2003 after recognition of extrapancreatic manifestations [5], and its name was officially coined in 2010 [6]. Since then, IgG4-RD has been described in most organ systems [7], including the retroperitoneum [2]. 

We present the case of a 64-year-old woman with biopsy-proven isolated IgG4-related RPF, without other systemic manifestations of the disorder. In light of persistent renal dysfunction due to obstructive uropathy, she was treated with a short course of corticosteroids and rituximab, which was followed by improvement in kidney function. We review the existing literature on IgG4-related RPF and the potential role of B-cell depletion with rituximab as primary treatment for this systemic disorder. 

## Case report 

**Figure 1.**
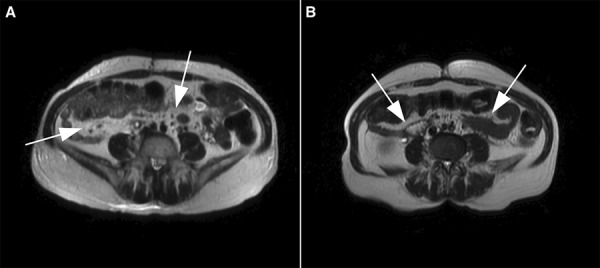
Axial T2-weighted magnetic resonance imaging (MRI) of the pelvis showing the amount of soft tissue before (A) and after (B) therapy. A: There is increased soft tissue in the retroperitoneum inferior to the aortic bifurcation, the cause of the bilateral ureteral obstruction. B: The amount of soft tissue in the retroperitoneum distal to the aortic bifurcation has markedly decreased. There is mild reduction in the right hydronephrosis and significant reduction in the left hydronephrosis. White arrows point to areas of retroperitoneal fibrosis.

A 64-year-old woman with a past medical history of hypertension and right hemicolectomy due to colonic adenomas presented to an outside hospital with a 3-month history of dry heaves and malaise. Prior to this illness, the patient had been in her usual state of health. At the outside hospital, she was found to have acute kidney injury with a serum creatinine of 21 mg/dL (baseline of 1.3 mg/dL). She required emergent hemodialysis for kidney failure and hyperkalemia. A CT scan of the abdomen and pelvis without contrast was performed, demonstrating bilateral hydronephrosis with no evidence of a mass or stone. A retrograde pyelogram was performed, demonstrating bilateral ureteral obstruction with medial deviation of the ureters, and she subsequently underwent bilateral ureteral stents placement. She was discharged with a serum creatinine of 2.7 mg/dL. Four weeks later, she presented to our hospital after she had noticed passing one of the stents in the urine. At that time, her serum creatinine was 3.1 mg/dL. A CT scan of the abdomen and pelvis without contrast showed moderately-severe bilateral hydronephrosis, and absence of the right ureteral stent. It also demonstrated nonspecific presacral and retroperitoneal fat stranding (Figure 1). A retrograde pyelogram revealed diffuse mucosal irregularity of the right ureter (Figure 1). A new stent was placed in the right ureter. To further investigate the CT scan finding, an MRI of the abdomen and pelvis was performed, demonstrating increased soft tissue in the retroperitoneum inferior to the aortic bifurcation, which was thought to be the cause of her ureteral obstruction (Figure 1). An extensive workup for RPF was unrevealing, including negative urine cytology, nonreactive ANA and ANCA antibody, and absence of monoclonal proteins on serum and urine protein electrophoreses. However, the ESR was elevated at 73 mm/h, and the serum IgG4 level was elevated at 259 mg/dL (normal 8 – 140 mg/dL). The differential diagnosis at that time included retroperitoneal fibrosis and malignancies such as lymphoma. She was discharged home after improvement of her serum creatinine to 2.2 mg/dL. 

**Figure 2.**
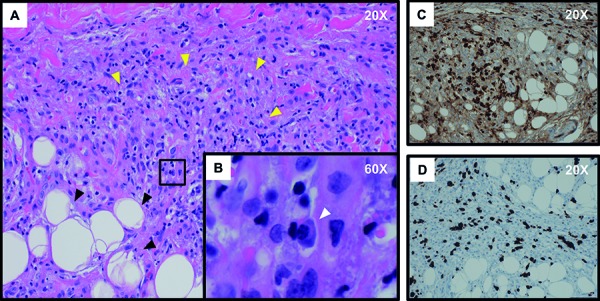
Retroperitoneal biopsy findings. A: Hematoxylin and eosin staining showing fibrous proliferation with hyalinized collagen, focally-forming storiform fascicles, dense lymphoplasmacytic infiltrate (yellow arrowhead) with occasional germinal centers. Black arrowheads showing the normal retroperitoneal fibro-adipose tissue. B: High-power field showing classical plasma cell (white arrowhead) within the infiltrate; immunohistochemical staining assessment of the IgG4/IgG ratio performed by quantification of stained cells, showing IgG-positive lymphocytes and plasma cells of 60 – 70/high-power field (C), and IgG4-positive lymphocytes and plasma cells of 30/high-power field (D), demonstrating an IgG4/IgG ratio of 42 – 50%.

Six days later, she underwent surgical bilateral ureterolysis, right ureteral reimplantation, and lysis of extensive bowel adhesions. During the procedure, the retroperitoneal soft tissue density was biopsied, which revealed fibrous proliferation with hyalinized collagen, focally-forming storiform fascicles, dense lymphoplasmacytic infiltrate with occasional germinal centers (Figure 2A, B), and focal obliterative phlebitis. Immunohistochemical staining revealed IgG4-positive plasma cells (60 – 70/high-power field), and the IgG4/IgG plasma cell ratio was > 40% (Figure 2C, D), in support of a diagnosis of IgG4-RD. Following the procedure, the serum creatinine dropped from 4.8 to 1.8 mg/dL. 

Two weeks later, she was started on a course of prednisone 40 mg once daily for 4 weeks followed by a taper. At the time, the serum creatinine was 1.57 mg/dL with an estimated GFR of 34 mL/min, placing her at stage-3b chronic kidney disease. The urinalysis revealed 2+ blood, 3+ protein, and no leukocytes. Her ESR and serum IgG4 level remained persistently elevated at 84 mm/h and 167 mg/dL, respectively. Complement factors C3 and C4 were normal at 140 mg/dL and 39 mg/dL, respectively. Her IgG4-RD responder index, a recently-developed disease activity score [8], was calculated at 12, which assumed that the RPF was persistent with involvement of two organs (retroperitoneum and kidneys), and that the disease activity was symptomatic, urgent, and damage was present. The random urine albumin-to-creatinine ratio was 1,650 mg/gm, suggestive of potential intrinsic renal disease, including tubulointerstitial nephritis. 

**Figure 3. Figure3:**
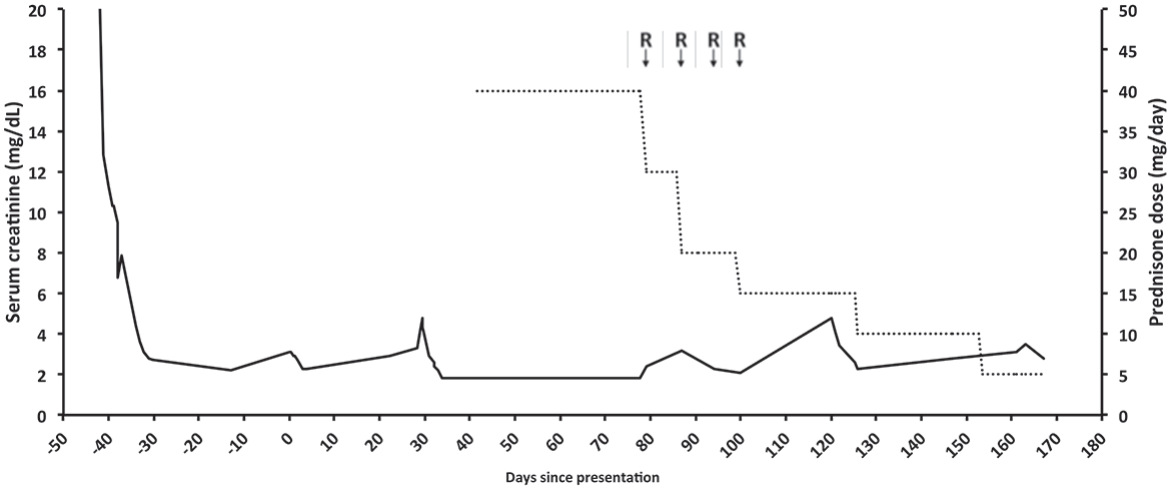
Time course of the serum creatinine and daily prednisone dose. “R” represents a 500-mg intravenous dose of rituximab.

She received a course of rituximab to abbreviate her course of glucocorticoids to 2 months. The hepatitis B surface antigen and the hepatitis B surface and core antibody titers were nonreactive. Five weeks after initiation of the prednisone, she received her first of 4 weekly infusions of rituximab 375 mg/m^2^, with a total cumulative dose of 2 g over 1 month. Prior to her first infusion of rituximab, the serum creatinine was 2.4 mg/dL, the ESR was 110 mm/h, the CRP level was 42 mg/dL (normal, < 0.8 mg/dL), and the serum IgG4 level had dropped to 94 mg/dL. One month after completing the rituximab course, her repeat serum creatinine was 2.3 mg/dL, the CRP normalized to 0.47 mg/dL, the serum IgG4 level further declined to 64 mg/dL, and the random urine albumin-to-creatinine ratio dropped to 370 mg/g. A repeat MRI revealed a substantial decrease in the amount of retroperitoneal soft tissue distal to the aortic bifurcation, which was accompanied by a decrease in the hydronephrosis bilaterally. The patient subsequently underwent the sequential removal of the two ureteral stents. 21 weeks after initiating therapy, the serum creatinine stabilized at 2.8 mg/dL, suggesting residual stage 4 chronic kidney disease. At the time, the serum IgG4 level was 49 mg/dL, the CRP was 0.24 mg/dL, and the ESR was 25 mm/h. The IgG4-RD responder index dropped to 3, consistent with a marked improvement in disease activity. We further assessed for response to B-cell depletion therapy with rituximab by performing peripheral blood flow cytometry, which showed a CD45-positive absolute count of 1.3K cells/µL (normal 1.00 – 3.33K cells/µL) with 0% CD19-positive cells (normal 4.6 – 22%), and 0% CD20 positive cells (normal 5.0 – 22.3%), in support of a favorable treatment response.[Fig Figure3]

## Discussion 

Here, we describe a case of IgG4-related RPF presenting with kidney failure due to extraluminal ureteral obstruction requiring ureteral stent insertion and surgical ureterolysis, and treatment with a short course of glucocorticoids and a 4-week course of rituximab. Two months following treatment, there was substantial decrease in the amount of retroperitoneal soft tissue, accompanied by a decrease in the hydronephrosis, and stabilization of the kidney function. 

A review of the literature identifies 45 cases of RPF in the setting of IgG4-RD, published in case series and individual case reports (Table 1) [9, 10, 11, 12, 13]. In brief, patients presented with an average age of 56 years, and 69% were men. Although not reported consistently, the serum creatinine was elevated in ~ 63% of patients at the time of the initial presentation, with an average value of 5.6 mg/dL. Most patients had evidence of perivascular and/or renal hilar retroperitoneal masses on a CT scan. 

In all cases, first-line therapy consisted of oral prednisone. The dose ranged from 10 to 75 mg/day, and the tapering regimen and treatment duration were variable, ranging from 2 months to long-term low-dose corticosteroids after the initial taper. Second-line treatment options used in the described cases of IgG-4 related RPF included mycophenolate mofetil [12], methotrexate [12], and azathioprine [10]. 

To date, there is no consensus regarding the optimal therapy of IgG4-RD, and expert opinion is derived from observational studies and uncontrolled single-arm trials. The mainstay of therapy is the use of glucocorticoids as monotherapy. Clinical response to glucocorticoids is observed in most patients initially with improvement in symptoms, which is accompanied by a reduction in IgG4-related masses and serum IgG4 levels. Due to the heterogeneity of the disease, an IgG4-RD Responder Index was developed to monitor response to therapy. This index incorporates symptoms, organ damage, urgency of care, and the serum IgG4 level. Although most patients initially respond to glucocorticoids, the caveat to monotherapy with glucocorticoids includes relapse and disease progression in most cases, long-term steroid-related toxicity, and heterogeneity of treatment response to glucocorticoids in patients, varying from a few weeks to several months. As such, some experts in the field have advocated for adding a steroid-sparing agent to complement the therapy and avoid these concerns. This includes the use of B-cell depletion therapy with the anti-CD20 antibody, rituximab. The efficacy of rituximab has been evaluated in a prospective open-label trial of 30 patients with IgG4-RD in whom 3 (10%) patients had retroperitoneal fibrosis and 2 (7%) had aortitis [14]. Participants were treated with 2 doses of rituximab (1,000 mg each) 2 weeks apart. If patients were not steroid naïve, they were required to discontinue the medication within 2 months of receiving rituximab. Disease response occurred in 97% of participants, as evidenced by drop in serum IgG4 levels and a progressive improvement in the IgG4-RD Response Index. The primary outcome of a decline in the IgG4-RD Responsiveness Index of 2 points or greater, the absence of disease flare before month 6, and no glucocorticoid use between month 2 and month 6 was achieved in 77% of patients. 47% were in complete remission at 6 months, and 40% remained in complete remission at 12 months. The authors concluded that rituximab appeared to be effective in treating IgG4-RD, even without concomitant use of glucocorticoid therapy. A randomized controlled trial would be required to validate the findings of this pilot single-arm trial and determine the appropriate role of B-cell depletion therapy in IgG4-RD. In our patient, we opted to use a rituximab dose of 375 mg/m^2^ every week for a total of 4 doses. We observed an improvement in the IgG4-RD Responder Index and normalization of the serum IgG4 level and markers of inflammation. While the observed decrease in the amount of retroperitoneal soft tissue and hydronephrosis as well as the improvement in the kidney function are coincidental, we can only speculate whether the rituximab shortened the natural history of the IgG4-related RPF by promoting the removal of B-cells and accelerating the resolution of the retroperitoneal inflammation. 

In a systematic review, the efficacy of monotherapy with glucocorticoids was examined in 1,220 patients with IgG4-RD, of whom 97% had a therapeutic response. However, relapses were reported in 33% of patients [15]. In a retrospective cohort study of 60 patients with IgG4-RD treated with rituximab of whom 68% were treated without glucocorticoids, 95% had a clinical response to rituximab, and 37% experienced relapses following treatment at a median follow-up of 244 days [16]. In this retrospective study, the baseline serum IgG4 level, the serum IgE level, and the eosinophil count predicted independently future risk of relapse [16]. 

When considering rituximab for IgG4-related RPF where its role has not been established, potential risks of the therapy need to be considered, including bone marrow toxicity leading to immunosuppression, hepatic toxicity, and neuropathy, and as a result, until further informative clinical studies become available, decision making should be carefully individualized. 

In conclusion, rituximab monotherapy can be used to induce and maintain remission in patients with IgG4-related RPF. In this case report, the use of rituximab provided a successful means of steroid-sparing with maintenance of remission and subsequent improvement in renal function. Whether this remission is long-lasting or temporary requires further follow up. Future studies should explore the degree of suppression of the fibrosis at a molecular level, establish appropriate dosing for the therapy, and examine the role of rituximab in other manifestations of IgG4-RD. 

## Funding 

The authors received no specific funding for this work. 

## Conflict of interest 

The authors declare no conflict of interest. 

**Table 1.** Summary of the clinical, imaging, laboratory, and treatment features of patients with IgG4-related retroperitoneal fibrosis.

Authors [reference]	Patient #	Age	Gender	Clinical presentation	CT scan findings	Serum creatinine (mg/dL)	CRP (mg/dL)	ESR (mm/h)	Serum IgG4 (mg/dL)	IgG4:IgG ratio (%)	Treatment	Duration of therapy
Chiba et al. [9]	1	75	F	Swelling of lacrimal glands	NR	0.6	0.10	NR	508	NR	Prednisolone 30 mg/day	Indefinite
	2	62	M	Fever, joint pain	Soft tissue mass in left renal hilus with hydronephrosis	1.2	20.00	NR	320	NR	Prednisolone 10 mg/day	
	3	60	F	Swelling of salivary glands, dry mouth	NR	0.6	0.10	NR	861	NR	Prednisolone 40 mg/day	
	4	63	M	Visual disturbance	NR	0.8	0.20	NR	433	NR	Prednisolone 35 mg/day	
	5	75	M	Back pain	Well-defined periaortic soft tissue mass and left renal hilum mass	1.0	1.10	NR	240	NR	Prednisolone 30 mg/day	
	6	69	F	Breast lump (later diagnosed as breast cancer)	Periaortic mass extending to the hilum of both kidneys	1.1	0.60	NR	143	NR	Prednisolone 50 mg/day	
	7	74	F	Swelling of lacrimal glands	NR	0.5	0.00	NR	1,270	NR	NR	
	8	79	M	Edema of the lower extremities	NR	1.1	0.70	NR	188	NR	Prednisolone 30 mg/day	
	9	73	M	Visual disturbance	NR	0.7	1.90	NR	1,790	NR	NR	
	10	71	M	Dyspnea	NR	0.7	0.70	NR	600	NR	NR	
Koo et al. [10]	1	59	F	Chest discomfort	Retroperitoneal mass with the largest measuring an average of 4.3 cm in diameter	NR	1.06	31	NR	46	Prednisone 40 mg/day	8 months
	2	75	M	Chest discomfort		NR	5.26	120	NR	76	Prednisone 60 mg/day	2 months
	3	62	M	Abdominal pain		NR	8.14	58	NR	42	Prednisone 60 mg/day	2 months
	4	56	M	Oliguria		NR	2.59	105	NR	41	Prednisone 75 mg/day	5 months
	5	55	F	Abdominal pain		NR	0.67	52	NR	42	Prednisone 50 mg/day	8 months
	6	65	M	Left flank pain		NR	0.97	33	NR	87	Prednisone 60 mg/day	9 months
	7	43	F	Generalized edema		NR	1.11	47	NR	66	Prednisone 60 mg/day	10 months
	8	64	M	Asymptomatic		NR	0.10	16	NR	62	NR	NR
	9	56	F	Right flank pain		NR	0.10	7	NR	55	NR	NR
Fernández-Codina et al. [11]	n = 24	Average of 53	M:F = 19:5	Pain (79%), constitutional (38%), vascular structure compromise (33%), and hydronephrosis (71%)	NR	Elevated in 64% of patients (average of 5.6)	Elevated in all patients	Elevated in 94% of patients (average of 73)	NR	Elevated (> 40%) in 25% of patients	Prednisone 1 mg/kg/day for 1 month followed by a taper over 24 months; other treatments included mycophenolate mofetil and tamoxifen	25 months (20 patients) Indefinite (4 patients)
Niaz et al. [12]	1	46	M	Low back pain	Diffuse circumferential soft tissue mass encasing the infrarenal abdominal aorta up to bifurcation of iliac vessels, with entrapment of both ureters	5.7	74.90	62	NR	70	Prednisone 1 mg/kg for 3 days, followed by 5 mg/day for 3 months; methotrexate 10 mg weekly	Indefinite
Monti et al. [13]	1	54	F	Diffuse arthralgia	Perivascular ulcerative mass involving the aortic arch and solid lesion surrounding the splenic artery with splenic thrombosis and splenomegaly	NR	NR	NR	599	Elevated	Low-dose prednisone	Indefinite

NR = not reported.
